# Lung injury after asphyxia and hemorrhagic shock in newborn piglets: Analysis of structural and inflammatory changes

**DOI:** 10.1371/journal.pone.0219211

**Published:** 2019-07-05

**Authors:** Birte Weber, Marc Robin Mendler, Ina Lackner, Alexander von Zelewski, Severin Höfler, Meike Baur, Christian Karl Braun, Helmut Hummler, Stephan Schwarz, Jochen Pressmar, Miriam Kalbitz

**Affiliations:** 1 Department of Traumatology, Hand-, Plastic-, and Reconstructive Surgery, Center of Surgery, University of Ulm, Ulm, Baden-Württemberg, Germany; 2 Division of Neonatology and Pediatric Critical Care, Department of Pediatrics and Adolescent Medicine, University of Ulm, Ulm, Baden-Württemberg, Germany; 3 Institute of Clinical and Experimental Trauma-Immunology, University Hospital of Ulm, Ulm, Baden-Württemberg, Germany; 4 Division of Neonatology, Department of Pediatrics, Sidra Medicine, Doha, Qatar; Auburn University College of Veterinary Medicine, UNITED STATES

## Abstract

**Objective:**

Asphyxia of newborns is a severe and frequent challenge of the peri- and postnatal period. The purpose of this study was to study early morphological, immunological and structural alterations in lung tissue after asphyxia and hemorrhage (AH).

**Methods:**

44 neonatal piglets (age 32 hrs) underwent asphyxia and hemorrhage (AH) and were treated according to the international liaison committee of resuscitation (ILCOR) guidelines. For this study, 15 piglets (blood transfusion (RBC) n = 9; NaCl n = 6, mean age 31 hrs) were randomly picked. 4 hours after ROSC (return of spontaneous circulation), lung tissue and blood samples were collected.

**Results:**

An elevation of myeloperoxidase (MPO) activity was observed 4 hrs after AH accompanied by an increase of surfactant D after RBC treatment. After AH tight junction proteins Claudin 18 and junctional adhesion molecule 1 (JAM1) were down-regulated, whereas Occludin was increased. Furthermore, after AH and RBC treatment dephosphorylated active form of Connexin 43 was increased.

**Conclusions:**

AH in neonatal pigs is associated with early lung injury, inflammation and alterations of tight junctions (Claudin, Occludin, JAM-1) and gap junctions (Connexin 43) in lung tissue, which contributes to the development of lung edema and impaired function.

## Introduction

Asphyxia is a common phenomenon during birth and the postnatal period [1], which leads to the annual death of approximately 1 million newborns worldwide [2]. Asphyxia is described as a severe disturbance of oxygen supply of the fetus [3] with intrapartum pH of less than 7.00 and a base deficit exceeding -12 mmol/l [4]. Lung injury occurs in 25% of all asphyxiated newborns, which ranges from a mild respiratory distress to pulmonary hemorrhage/severe respiratory failure [5]. Alveolar edema is an early factor in the development of acute respiratory distress syndrome (ARDS), which results from an increase of the permeability of the alveolar epithelial barrier. Driving mechanisms for the breakdown of the epithelial barrier are neutrophil infiltration, inflammation, damage of epithelial and endothelial cells as well as disruption of tight junctions (TJ) between alveolar epithelial cells [6].

Pulmonary edema is associated with increased mortality, as described in the context of acute lung injury (ALI) in adults [7]. ARDS in newborns requires respiratory therapy which is associated with complications such as pneumothorax, pulmonary interstitial emphysema and pneumopericardium [8]. Furthermore, inflammatory influence in the developing lung is harmful due to impaired alveolarization postpartum which is associated with the development of future lung diseases [9]. In adults with ARDS alveolar macrophages are activated and release cytokines such as tumor necrosis factor (TNF) and interleukin 1 (IL-1). TNF has been shown to disrupt pulmonary TJ proteins [10]. Furthermore pulmonary influx of neutrophils has been correlated to lung injury and increased permeability of the alveolar-capillary membrane [11].

However, the mechanisms of ADRS in term newborns with AH are not fully understood. Therefore, the present study investigates the pulmonary consequences of AH in newborn piglets, treated in accordance with the ILCOR (International Liasion Committee of Resuscitation) with cardiopulmonary resuscitation (CPR), ventilation, epinephrine and with either red blood cell (RBC) transfusion or crystalloids (NaCl). The present investigation aimed to carve out the effect of different volume replacement regimes on lung damage after asphyxia with blood loss. Successful resuscitation was achieved in all animals independent of group assignment and without differences in time to ROSC between crystalloid and early transfusion [12]. In this study, we used the pig as a large animal model, because of the physiological and anatomical similarity to humans, especially in regard to the cardiopulmonary system. Furthermore, this model is well-established and previously published, for example by Mendler, et al. (2016) [13]. The current report suggests that pulmonary inflammation and tight junction alterations contribute to the development of pulmonal dysfunction after AH in newborn pigs.

## Materials and methods

### Animals

44 Piglets (age: 32 hrs, weighing 1.220 kg (1.060–1.495 kg)) underwent asphyxia and hemorrhage. The animal procedure was approved by the responsible government authority (Regierungspräsidium Tübingen, Germany; TV-No:1262 and 1320) in accordance with the guidelines of the Federation of European Laboratory Animal Science Association (FELASA). A detailed description of the animal experiment is already published in Mendler, et al. (2018) [12].

The animals were randomized into two therapy groups either with crystalloids (NaCl) or blood-transfusion. Sham animals underwent anesthesia without AH. For further analysis of the lung tissue 21 animals were randomly picked: 9 RBC, 6 NaCl and 6 sham.

### Anesthesia and preparation

Animals were anesthetized with propofol/fentanyl and orotracheally intubated with uncuffed ET tubes. Ventilation was conducted pressure controlled with a FiO_2_ of 0.30, a PEEP of 5 cm H_2_O, inspiratory time of 0.4 s and an initial ventilation rate of 20/min. Ventilation frequency was adapted to a PaCO_2_ of 35–45 (normocapnia). The body-temperature was maintained at 39.0–39.5°C using a heating mattress and an overhead warmer.

A double lumen arterial line was inserted into the femoral artery for continuous blood pressure measurements and to obtain arterial blood gas analyses (ABL 800, Radiometer, Willich, Germany) as well as blood samples (serum, plasma). Additionally, a double lumen venous line was placed into the femoral vein for central venous pressure (CVP) measurements, i.v. drug and fluid application and maintenance fluids.

### Asphyxia

After initiation of anesthesia, the FiO_2_ decreased to 0.21 and after a 15 min baseline blood samples were collected (serum and plasma). Hypoxia was induced by reduction of FiO_2_ to 0.08 and simultaneous exposure to hypercapnia (FiCO_2_ = 0,07). Alveolar hypoventilation was induced step by step by reducing the ventilation frequency every 10 min by 10/min. A continuous blood loss (2 ml/kg/min) from the arterial line was induced and continued until asystole occurred. We defined a cardiovascular arrest as the loss of pulse (blood pressure < 10 mmHg) or the loss of regular ECG activity (asystole).

### Resuscitation

Resuscitation was performed in line with the ILCOR guidelines. A FiO_2_ of 0.21 was initially used. After 30 sec of adequate respiratory support, chest compression was initiated with a rate of 90/min. Inflations were interposed to chest compression using a 3:1 ratio and a two-thumb-technique. Resuscitation was implemented by a trained team and CPR was always given by the same team member. Chest compression depth was aimed to generate a systolic pressure of 50 mmHg.

Success of the resuscitation was evaluated every 30 seconds. 90 seconds after the begin of asystole, FiO_2_ was increased to 1.0 and epinephrine (20 μg/kg) was given. CPR was continued. After 120 seconds of resuscitation, piglets underwent either a transfusion of 10 ml/kg of NaCl 0.9% (n = 6) or transfusion of their withdrawn blood (n = 9). Epinephrine bolus was repeated every 3 min. The return of spontaneous circulation (ROSC) was defined as a heart rate (ECG) of > 60 bpm and a measurable arterial blood pressure curve. 6 animals underwent sham procedure, which included anesthesia and catheterization without AH.

One hour after ROSC, the NaCl group received an additional transfusion of 10 ml/kg of NaCl over 15 min. Hemodynamic parameters were continuously monitored for 4 hrs and the observation period was terminated by intravenous potassium chloride.

### Sample collection

Serum and plasma samples were collected at baseline and 4 hrs after ROSC and kept on ice. After centrifugation (800 g for 5 min at 4°C followed by 13000 g for 3 min at 4°C), serum and EDTA-plasma were removed and stored at -80°C until analysis. Lung tissue samples were obtained 4 hrs after ROSC. The upper lobe of the right lung was fixed with 4% formalin, followed by embedding in paraffin. The upper lobe of the left lung was quick-frozen in liquid nitrogen, followed by storage at -80°C until analysis.

### Calculation of the oxygenation ratio (Horovitz)

The Horovitz oxygenation ratio was calculated as the arterial oxygen partial pressure divided by the fractional inspired oxygen. The values were determined by means of an arterial blood gas analysis at baseline, at asystole, at ROSC and after 4 hrs.

### Surfactant protein D-ELISA

For determining plasma Surfactant protein D levels, sandwich-ELISA Pig SFTPD / Surfactant Protein D ELISA kit (LifeSpan BioSciences Inc., Seattle, WA, USA) was used following the manufacturer’s recommended protocol. All results were normalized to the respective amount of total plasma protein using a Pierce Bicinchoninic Acid (BCA) Assay (Thermo Fisher Scientific, Waltham, MA, USA).

### Western blotting

Tissue of the left lung obtained 4 hrs after resuscitation or sham procedure was homogenized and lysed by using 1x RIPA Lysis Buffer (Thermo Fischer, Rockford, IL, USA), containing complete Mini-protease inhibitor and PhosSTOP protease inhibitor cocktail (Roche, Indianapolis, IN, USA). Protein concentrations of the homogenates were determined by using Pierce BCA Protein Assay Kit (Thermo Scientific). The samples were loaded under reducing conditions onto a 4–20% Mini-Protean TGX precasted gels (Bio-Rad Laboratories, Munich, Germany). After electrophoresis, proteins were transferred by a Trans-Blot Turbo Transfer System using Mini Transfer Packs (both from Bio-Rad). The blots were blocked with 5% milk and then incubated with different primary antibodies (see below) overnight at 4° C. Following antibodies were used: Rabbit anti-C5aR1 (Proteintech, Manchester, UK), Rabbit anti-C5aR2 (abcam, Cambridge, UK); anti-TNF alpha antibody (abcam, Cambridge, UK), anti-IL-6 antibody (abcam), Rabbit anti-Connexin 40 (GeneTex, Irvine, CA, USA), and Rabbit anti-Cx43 (Cell Signaling Technology, Danvers, MA, USA).

After washing, HRP-anti-rabbit antibody (Cell Signaling Technology, Danvers, MA, USA) was used as secondary antibody at room temperature for 1 h. Chemiluminescent HRP Hy Glo (Denville Scientific Inc, South Plainfield, NJ, USA) was used for developing. The blots were analyzed by the ChemiDoc (BioRad Laboratories GmbH, Munich, Germany) and the Image Lab Software (Version 5.2, BioRad). By using the stain-free imaging technology of BioRad, a normalization of the quantitative western blot data to the total protein amount in each lane was conducted. The presented data were normalized C5aR1 and C5aR2 protein amounts.

### Immunohistochemistry (caspase-3, JAM-1, surfactant D) and hematoxylin and eosin (H.E). staining

For immunohistochemistrial staining, formalin-fixed and paraffin embedded right lungs were analyzed. As primary antibodies anti-Caspase-3 (Cell Signaling Technologies, USA), Anti-JAM-1 (biorbyt, Cambridge, UK) and anti-Surfactant-D (Bio-Rad AbD Serotec Limited, UK) were used. Dako REAL Detection System (Dako, Glostrup, Denmark) constituted the secondary system. Signal density of stained protein was measured in 3 randomly chosen, distinct fields of vision (100x magnification) from each slide due the Axio Imager M.2 microscope and the Zeiss ZEN 2.3 software (Zeiss, Jena, Germany). Results are presented as mean density of each group (arbitrary units). For evaluation of histolomorphological changes, a modified lung score was used. H&E (Morphisto, Frankfurt am Main, Germany) stained sections were analyzed for 1) neutrophilic infiltration, 2) formation of hyaline membranes, 3) shedding of alveolar epithelial cells and 4) intra-alveolar hemorrhage. Items were scored: 0 for no visible changes, 1 for rare, occasional changes, 2 for moderate changes, 3 for severe changes and 4 for very severe, widespread damage. For each section, 10 fields of vision (magnification: 400x) were scored and mean values were calculated for each item respectively and added to yield a final score value for each animal. All sections were scored in a blinded manner.

### Immunofluorescence staining (Occludin, Claudin) and TdT-mediated dUTP-biotin nick end labeling (TUNEL)

For immunofluorescence (IF) (Occludin, Claudin 18), the paraffine embedded samples were dewaxed and rehydrated. Subsequent endogenous peroxidase-activity was blocked using a 3% H_2_O_2_ solution. The sections were incubated at 4° C overnight with the following primary antibodies: anti-Occludin (polyclonal anti-Occludin-antibody, unconjugated, bs-1495R; Bioss Antibodies Inc., USA), anti-Claudin 18 antibody (34H14L15; ABfinity Rabbit; Thermo Fisher Scientific, USA). As secondary antibody Alexa Fluor 488 (Thermo Fisher Scientific, USA), was used. Nuclear counterstain was applied (Hoechst 33342; Thermo Fisher Scientific, USA) and sections mounted with ProLong Gold Antifade (Thermo Fisher Scientific, USA) for long-term preservation. Imaging was performed using Axio ImagerM.1 microscope and the Zeiss AxioVision software 4.9 (Zeiss). Evaluation of fluorescence intensity was conducted with the Software Image J1.

TUNEL staining was performed by using CF 488 TUNEL Apoptosis Detection Kit (Biotium, Fremont, CA, USA). A counterstain with Höchst 33342 (Sigma, Darmstadt, Germany) was done. Results of TUNEL positive nuclei were presented in percentage of whole nuclei number.

### Lung myeloperoxidase (MPO)

Left upper lung lobe tissue was homogenized (Ultraturrax T25; Jahnke und Kunkel, Staufen, Germany) in a buffer containing 10.35 g KH_2_PO_4_ (Merck Milipore, Germany) in 950 ml distilled water adjusted to pH 5.4 using 0.91 g K_2_HPO_4_ (Merck Milipore, Germany) in 50 ml distilled water and 0.5% hexadecyltrimethylammonium bromide (Sigma-Aldrich, Germany). Tissue homogenates were incubated at 60° C for 2 hrs followed by centrifugation.

The tissue was mixed with tetramethylbenzidine (Sigma-Aldrich, Germany) and 0.002% H_2_O_2_ (Fluka, Germany), followed by an incubation at 37°C. Extinction values were read at 450 nm. MPO activity was calculated on the basis of standard curves. Thereafter, perchloric-acid precipitation was performed and the protein concentration was determined using the Pierce BCA Protein Assay Kit (Thermo Fisher Scientific, USA).

### Statistical procedures

All values were expressed as mean ± SEM. Data were analyzed by one-way ANOVA followed by Dunnett’s or Tukey’s multiple comparison test. p≤0.05 was considered statistically significant. GraphPad Prism 7.0 software was used for statistical analysis (GraphPad Software, Incorporated, San Diego, Ca, USA).

## Results

### Lung injury after Asphyxia and Hemorrhage

**Fig 1:**
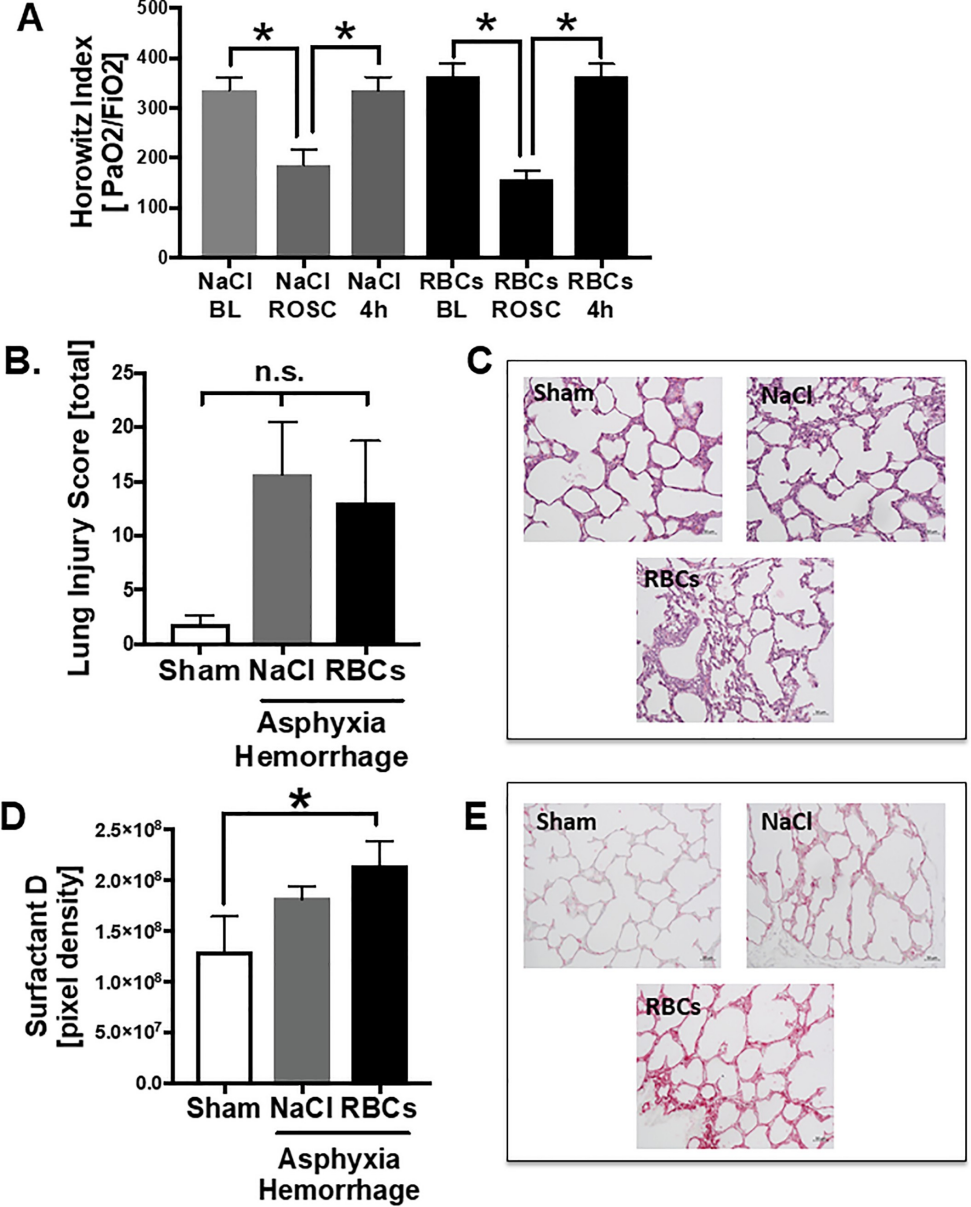
Lung injury after AH. A) Horovitz oxygenation index calculated as ratio of arterial oxygen partial pressure and the fractional inspired oxygen at baseline (BL), ROSC (return of spontaneous circulation) and 4 hrs after asphyxia. B) Lung injury score determined in H.E. stained lung tissue 4 hrs after asphyxia and hemorrhage (AH). C) Representative images of H. E. staining. D) Results of IHC staining of surfactant protein D in lung tissue. E) representative images of surfactant D staining. * p = 0.05, n.s. = not significant, graphical representation as mean ± SEM, NaCl n = 6, RBC n = 9, sham n = 6.

In Fig 1 the results of the analysis of lung injury are displayed. The clinical Horowitz score for oxygenation (Fig 1A), as well as the histological analysis of the lung tissue lung injury score (Fig 1B) revealed a tendency of lung damage after asphyxia and hemorrhage. The Horovitz score was significantly reduced at ROSC in both treatment groups, and moved back to baseline values 4 hrs after AH. One item of the histological injury score was the lung edema after asphyxia. The representative images of H.E. staining presented in Fig 1C illustrates infiltrations of cells in the lung tissue.

Surfactant D in lung tissue was increased only in the animals treated with red blood cells (RBCs) (Fig 1D), which is also shown in representative images of Surfactant D staining (Fig 1E).

### Local inflammation and activation of the complement system after AH

**Fig 2:**
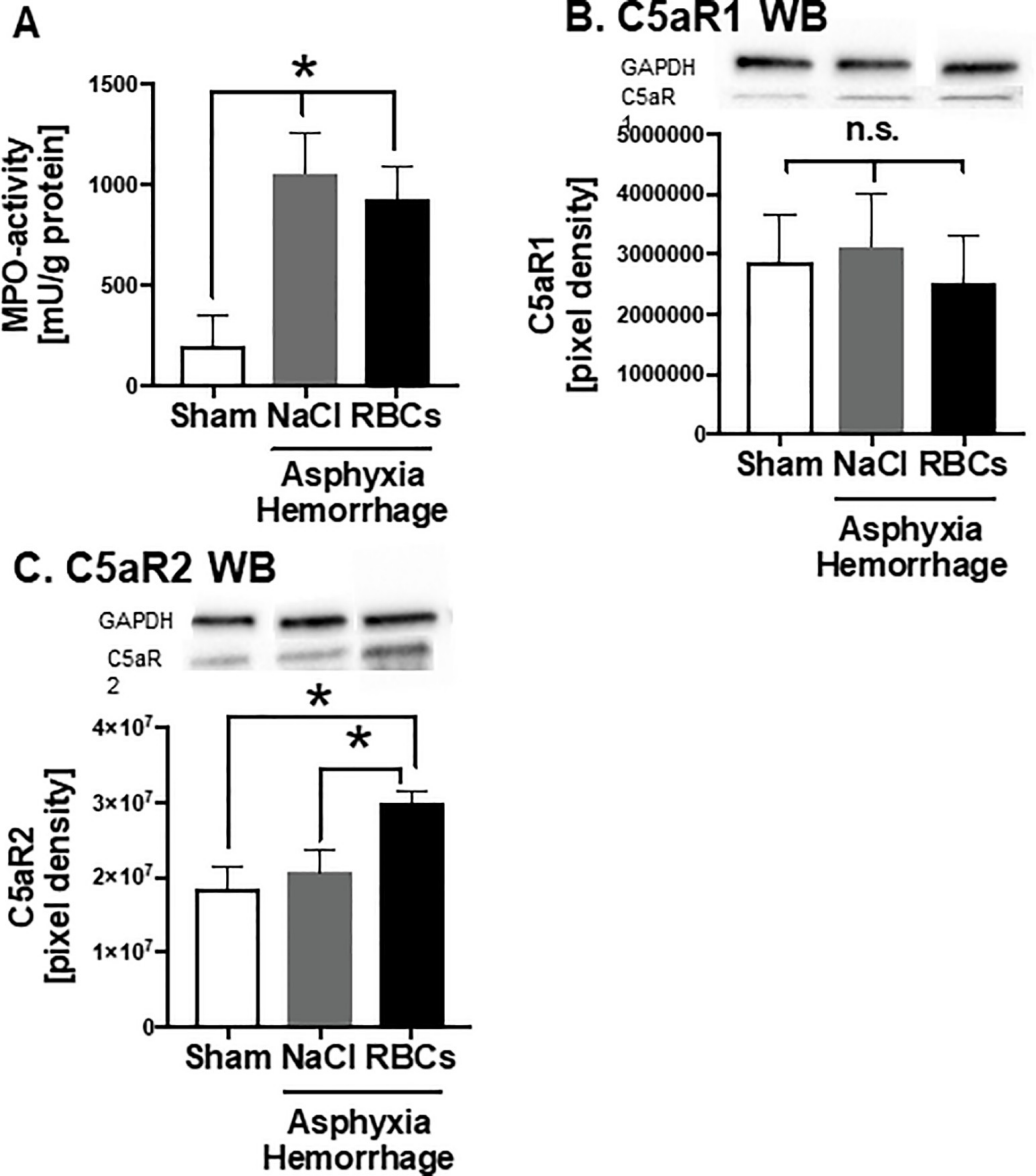
Local inflammation and activation of the complement system after AH. A) Myeloperoxidase (MPO) activity obtained from lung tissue samples. B) local amount of C5a receptor 1 and C) complement factor 5a receptor 2 protein in lung tissue normalized to the total protein amount of the samples, as well as representative western blots and loading control bands (GAPDH). * p = 0.05; n.s. = not significant, graphical representation as mean ± SEM, NaCl n = 6, RBC n = 9, sham n = 6.

Local inflammation might be an important mechanism for the development of lung tissue damage after asphyxia and hemorrhage. Therefore, appearance and activation of neutrophils in lung tissue was measured by MPO activity, which was increased in both treatment groups compared to sham treated animals (Fig 2A). Furthermore, we determined the activation of a complement system after asphyxia and hemorrhage by using western blot analysis. Whereas C5aR1 expression was not altered in both treatment groups compared to sham treated animals (Fig 2B), C5aR2 expression was significantly increased in lung tissue of animals with AH treated with red blood cells (Fig 2C).

### No elevation of apoptotic cells in lung tissue after AH

**Fig 3:**
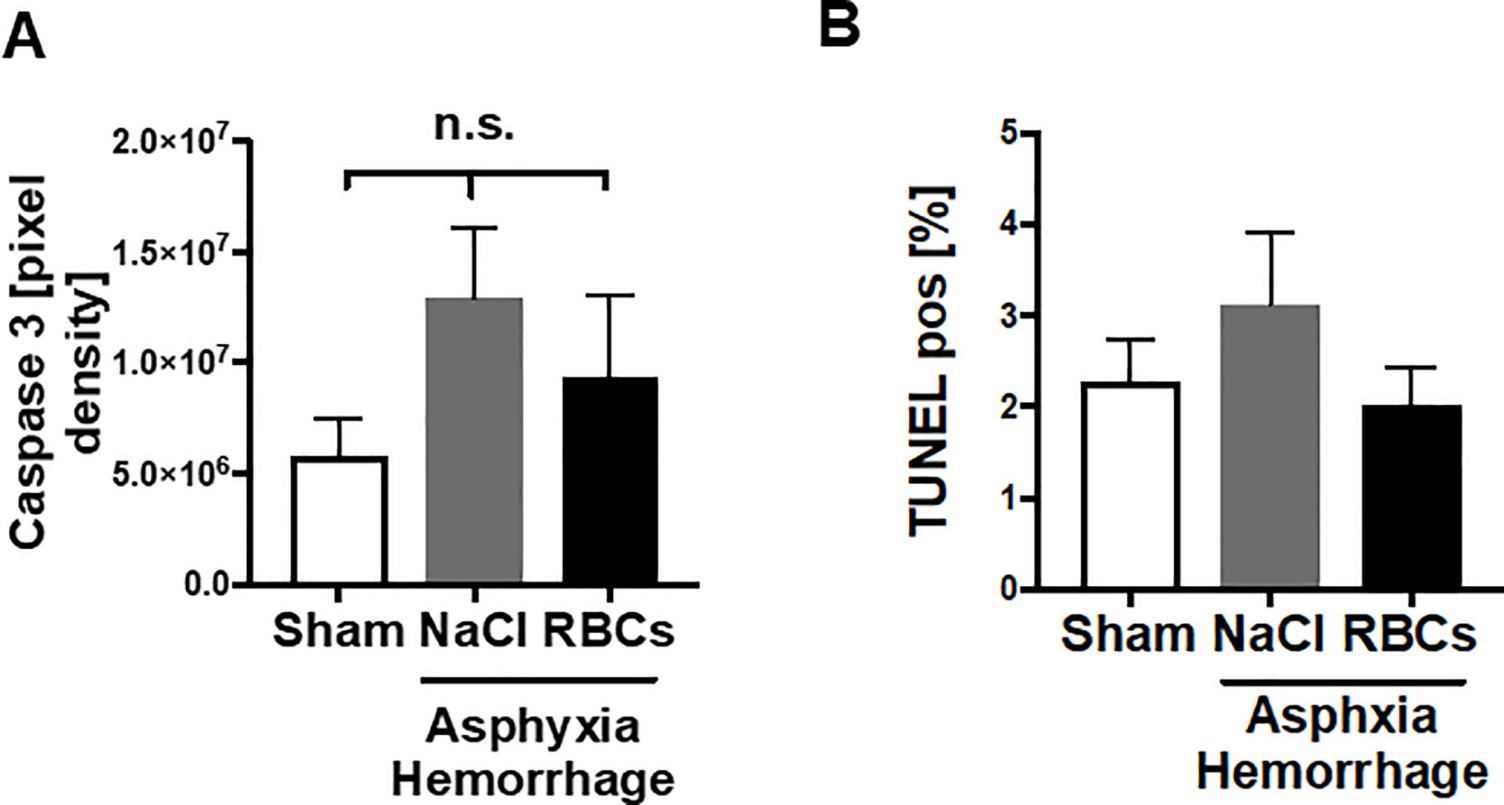
No elevation of apoptotic cells in lung tissue after AH. A) Results from caspase 3 analysis. B) TUNEL positive nuclei in percentage of whole cell count by DAPI staining; n.s. = not significant; graphical representation as mean ± SEM, NaCl n = 6, RBC n = 9, sham n = 6.

We measured apoptosis by two different methods: staining of caspase 3 in paraffin-embedded sections of lung tissue and the TUNEL assay. Neither caspase staining (Fig 3A) nor the determination of TUNEL (Fig 3B) positive cells, did show any significant differences between the treatment and sham groups at the respective time point in this study.

### Alterations in cell to cell contacts after AH

**Fig 4:**
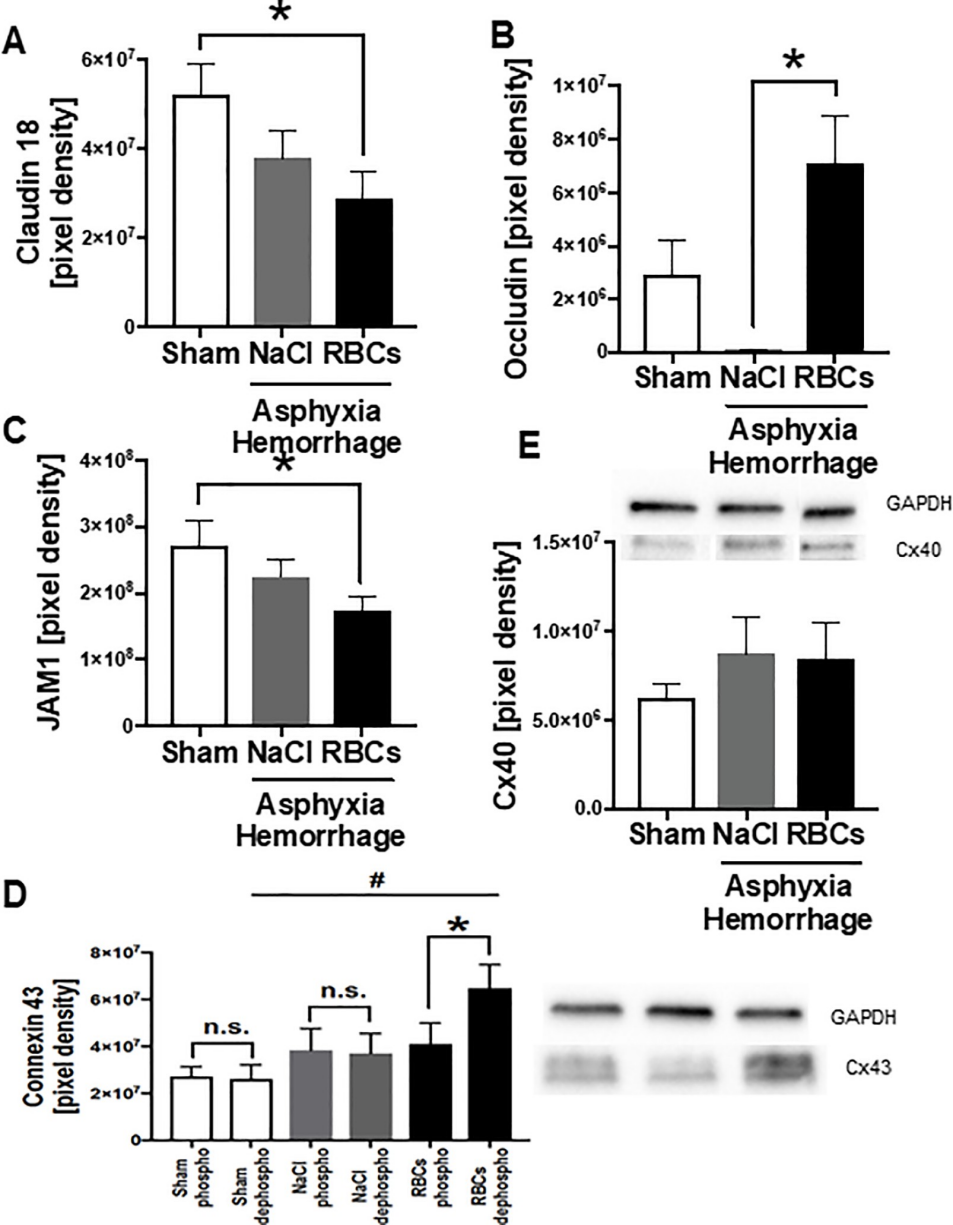
Alterations in cell cell contacts after AH. Results of IHC staining of A) Claudin 18, B) Occludin and C) JAM-1 of lung tissue after asphyxia. Alterations in gap junction protein amount of D) Connexin 43 and E) Protein amount of Connexin 40. * p = 0.05, n.s. = not significant, graphical representation as mean ± SEM, NaCl n = 6, RBC n = 9, sham n = 6.

In regard to cell-to-cell contacts, alterations between both treatment groups and sham treated animals were observed. The protein amount of the tight junction molecules Claudin-18 (Fig 4A) and JAM-1 (Fig 4C) were reduced in lung tissue after asphyxia and hemorrhage in the RBC treated animals, whereas Occludin was increased (Fig 4B). Red blood cell treated animals also showed changes in the gap junction protein Cx43. Compared to sham treated animals, an increase in dephosphorylated connexin proteins was observed by using western blot analysis (Fig 4D), while the Connexin 40 molecules did not differ between the sham and the treatment groups (Fig 4E).

## Discussion

In this study, we characterized early consequences of AH and different resuscitation protocols (RBC, NaCl) on lung injury.

To detect pulmonary function after asphyxia and hemorrhage, the literature describes different oxygenation indices in humans. Horovitz index was applied, which revealed significant differences at the time of ROSC and minimal changes 4 hrs after asphyxia compared to baseline. In our model re-transfusion of newborn blood instead of adult red blood cells was used. Therefore, there are differences in regard to fetal hemoglobin compared to the clinical situation in human neonates. In the neonatal blood higher concentrations of fetal hemoglobin are detected (71% +/- 7,7%) [14]. In healthy adults the proportion of fetal hemoglobin accounts for less than 2% (>18 years) [15], which has a stronger affinity to oxygen and therefore ensure a sufficient oxygen-supply during the fetal period [16].

All animals were mechanically ventilated during the observation period in the present study. Therefore, pulmonary alterations due to mechanical ventilation cannot be excluded when interpreting the observations in the present study.

The measurement of IL-6 and TNF in lung tissue did not present any changes as early as 4 hrs after asphyxia in both treatment groups (S1A and S1B) This result is in accordance to the observations of Mustafo, et al (2018) who described unchanged inflammatory markers IL-6, IL-8, TNF and IL-1ß in lung tissue of asphyxiated piglets [17]. In adults, cytokines were described as amplifier of lung injury and also as responsible for the recruitment of inflammatory cells into the lung [18].

In our study protocol whole blood re-transfusion was applied; therefor neutrophil activation due to contact to external surfaces followed by neutrophil extracellular trap (NET) formation may have occurred whereas in the common clinical setting of AH leucocyte depleted irradiated erythrocyte concentrates are transfused. In vitro activation of neutrophils by C5a leads to enhanced histone release, caused by neutrophil extracellular trap (NET)-formation [19]. In ARDS of humans extracellular histones were present in broncho-alveolar lavage fluid [20].

Complement anaphylatoxin C3a was found in the plasma of asphyxiated neonates, and an elevation between day 2 and 8 was associated with mortality [21]. Therefore, we investigated the complement-receptor expression in lung tissue after AH. Here, expression of C5aR1 was unchanged in both treatment groups whereas C5aR2 expression was significantly increased in RBC treated animals compared to NaCl treated animals and compared to controls. Genetic deficiency of either C5aR1 or C5aR2 markedly reduced the intensity of ALI in rodent model of lipopolysaccharide (LPS)-ALI, C5a-ALI and immunoglobulin G immune complex (IgGIC)-ALI [20]. Taken together, complement activation is involved in pulmonary inflammatory response to AH and resuscitation followed by resuscitation with re-transfusion of red blood cells.

In adult humans, blunt chest trauma leads to high serum levels of surfactant protein D (SP-D) 6 hrs, 24 hrs and 7 days after trauma and correlation between the SP-D levels and the injury severity score (ISS), as well as the development of complications was reported [22]. An elevation in serum SP-D-levels was also correlated with the mortality of patients receiving a mechanical ventilation and was described in the context of obstructive pulmonary disease, pneumonitis and pneumonia [23]. In the present study systemic SP-D levels were not significantly increased after AH compared to sham treated animals. This might be due to the early time point (4 hrs) after AH, which has to be further evaluated.

SP-D is a collagen-containing calcium dependent lectin named collectin involved in regulation of inflammation and innate immune response in lung tissue and is expressed by club cells and alveolar type II cells [24]. In the present study SP-D was locally elevated in RBC treated animals after AH. Nitrosylation of SP-D is associated with disruption of the mulitmeric structure and resulted in a pro-inflammatory signaling activity with macrophages [25]. Furthermore, collectins are involved in aggregating pathogens and the recruitment of neutrophils [26]. Taken together, SP-D up-regulation after AH in RBC group might occurs simultaneously with increased local MPO activity and C5aR2 expression and might therefore be due to pro- and anti-inflammatory mechanisms in the lungs.

In the present study, increased amount of dephosphorylated Connexin (Cx)43 protein in RBC treated animals after AH has been observed in lung tissue; while total Cx43 and Cx40 protein were unchanged.

On One hand, Cx43 is up-regulated in the state of hyperpermeability of endothelial monolayers treated with inflammatory mediators [27]. On the other hand inhibited Cx43-dependent communication reduced vessel hyperpermeability in a modal of airway instillation of hydrochloric acid [28] and reduced neutrophil recruitment into lung tissue after acute lung injury with [29]. Interestingly, in seawater aspiration caused p-PKC dependent Cx43 phosphorylation (p-Cx43), was associated with pulmonary inflammation and lung edema [30]. Therefore, Cx43 phosphorylation may contribute to increased vascular permeability and inflammation in the lung after AH and RBC transfusion.

Furthermore, tight junction alterations in alveolar epithelium are important in the pathogenesis of lung edema. In the present study JAM1 and Claudin 18 were downregulated 4 h after AH and RBC treatment whereas Occludin was up-regulated in RBC treated animals compared to NaCl treatment. Experiments with JAM-1 knock-out mice, as well as *in vitro* experiments with rat alveolar epithelial cells with depletion of JAM-1, determined a higher susceptibly for lung injury and formation of lung edema [31]. Furthermore, the loss of JAM-1 was accompanied with the downregulation of the tight junction molecule Claudin-18 [31]. JAM-1 knock-out mice furthermore presented a two-fold higher cellular infiltration in the lung, after endotoxin-treatment [31] which is in accordance with increased pulmonal MPO activity after AH observed in the present study. Further, Claudin-18 deficiency results in alveolar barrier dysfunction and impaired alveologenesis in mice. [32]. Therefore, reduced expression of either JAM1 and Claudin-18 may contribute to epithelial barrier dysfunction after AH in term-newborns.

Neonatal asphyxia is one of the main causes of acute respiratory distress syndrome in full-term newborns. In a rat model of intrauterine acute ischemic-hypoxia alveolar type II cells with positive surfactant type B staining were reduced. Furthermore, surfactant B protein and expression of surfactant A and B were reduced within the first 40 min after ischemia [33]. Mechanisms of neonatal ARDS are the combination of primary surfactant deficiency and surfactant degradation by plasma proteins leaking into the airways and local inflammation [34,35]. As presented in the S1C Fig, we measured a potential trend of systemic appearance of surfactant protein D after AH, which might be a systemic marker of lung damage after AH. To evaluate pulmonary cell death, we quantified apoptosis by caspase 3 and TUNEL staining. Here, we did not measure any significant elevation of apoptosis in lung tissue after asphyxia and hemorrhage, neither with the TUNEL staining kit nor with a caspase-3 staining. Here, we did not measure any significant elevation of apoptosis in whole lung tissue after asphyxia and hemorrhage, neither with the TUNEL staining kit nor with a caspase-3 staining. An antiapoptotic gene expression pattern was observed immediately after complete reoxygenation of asphyxiated mice. After 2.5 h, all pro- and antiapoptotic genes were downregulated [36]. In the literature, studies with increased apoptosis after hypoxia/ischemia [37] as well as studies which did not observe apoptosis [38] are reported.

## Conclusion

In line with other studies, we did not detect elevation of early inflammatory mediators in lung tissue, while the complement system was activated and also the presence of neutrophils was proven. Taken together, asphyxia and hemorrhage lead to impressive alterations of gap and tight junction molecules in lung tissue, which contribute to the development and aggravation of lung edema in newborn pigs.

## Supporting information

S1 Table ARRIVE guideline checklist of animal research. (PDF)

S1 Fig Results of TNF (S1A Fig) and IL-6 (S1B Fig) western blot 4 h after asphyxia and hemorrhage in newborn pigs with either NaCl (n = 6) or blood (RBC, n = 9) transfusion. Systemic levels of surfactant protein D in blood samples of pigs after asphyxia (S1C Fig), p = 0.05, n.s. = not significant, graphical representation as mean ± SEM.
(TIF)
