# Supplementary figures and images for: Lung injury after asphyxia and hemorrhagic shock in newborn piglets: Analysis of structural and inflammatory changes
Source: PLoS One. 2019 Jul 5;14(7):e0219211. doi: 10.1371/journal.pone.0219211 (PMC6611609; doi:10.1371/journal.pone.0219211)

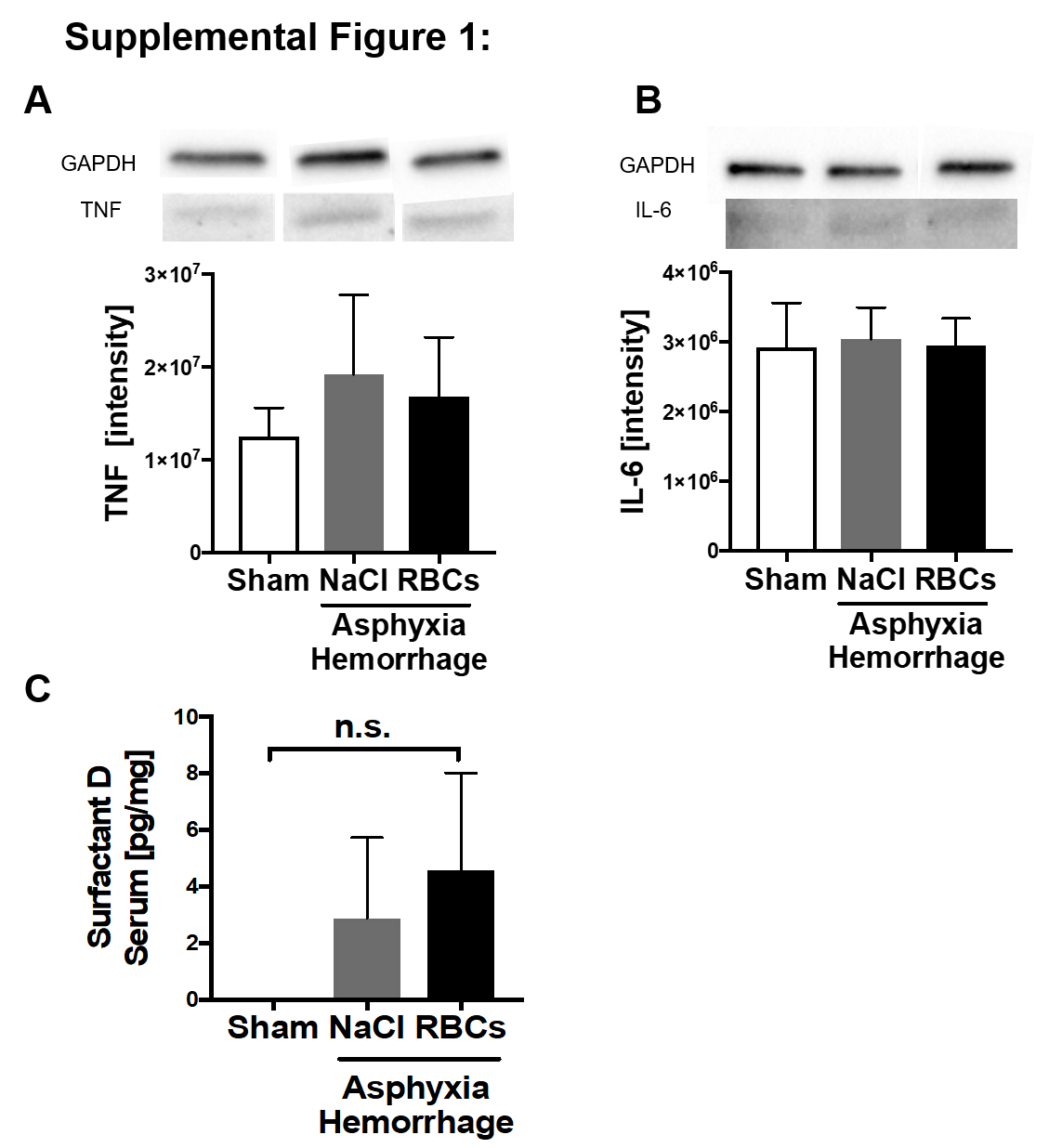

Supplement: S1 Fig — Systemic levels of surfactant protein D in blood samples of pigs after asphyxia (S1C Fig), p = 0.05, n.s. = not significant, graphical representation as mean ± SEM. (TIF) [file pone.0219211.s002.tif]
